# P-792. A Retrospective Study to Evaluate the Effectiveness of Oral Beta-lactam Antibiotics in Acute Bacterial Prostatitis

**DOI:** 10.1093/ofid/ofaf695.1002

**Published:** 2026-01-11

**Authors:** Yuichiro Nagase, Hidetoshi Nomoto, Shinya Tsuzuki, Norio Ohmagari

**Affiliations:** Japan Institute for Health Security, Shinjuku-ku, Tokyo, Japan; Japan Institute for Health Security, Shinjuku-ku, Tokyo, Japan; Japan Institute for Health Security, Shinjuku-ku, Tokyo, Japan; Japan Institute for Health Security, Shinjuku-ku, Tokyo, Japan

## Abstract

**Background:**

Fluoroquinolones (FQ) and trimethoprim–sulfamethoxazole (TMP–SMX) are first-line therapies for acute bacterial prostatitis, but rising antimicrobial resistance and adverse events limit their utility. Evaluating the efficacy of oral β-lactams (BL) may expand available treatment options.

**Table.**
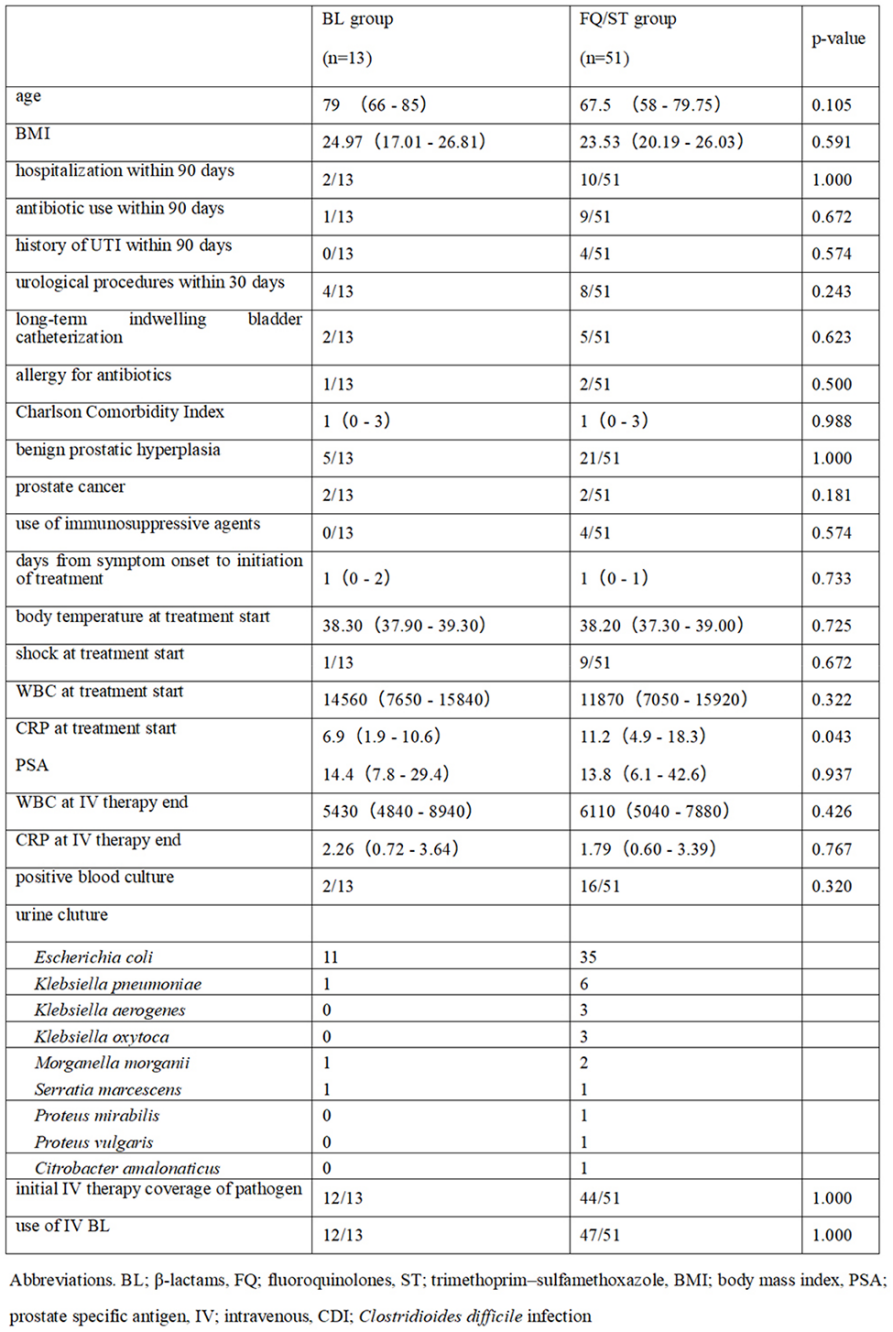
Patient Characteristics and Outcomes

**Methods:**

This retrospective study was conducted at the National Center for Global Health and Medicine, a tertiary hospital in Japan between 2013 and 2023. We included men aged 18 years or older who had received intravenous antimicrobial therapy for acute bacterial prostatitis based on medical records, were subsequently switched to oral therapy, and had *Enterobacteriaceae* as the cause. The primary endpoint was clinical cure, defined as improvement of symptoms, no recurrence of urinary tract symptoms within 30 days, no discontinuation or change of treatment due to worsening symptoms, and no adverse events. Secondary endpoints included *Clostridioides difficile* infection (CDI), sepsis, and all-cause mortality within 30 days of initiation of treatment. We compared the oral BL group with the FQ or TMP-SMX group (FQ/ST), receiving statistical analysis with AI support.

**Results:**

Of 337 registered prostatitis cases, 71 met inclusion criteria. Seven patients were excluded: two received other drugs and five were lost to follow-up. Finally, 13 patients who received BL and 51 received FQ/ST were included in the analysis. Baseline characteristics were comparable between groups (Table 1). There was no significant difference in the primary endpoint between the BL and FQ/ST groups (odds ratio: 0.255, 95% confidence interval: 0.065-1.005, p=0.056). Secondary endpoints were also not different for sepsis between the two groups; CDI and all-cause mortality did not occur in either group. TMP-SMX was frequently discontinued due to adverse effects, whereas recurrence occurred in both FQ and BL, even with susceptible isolates.

**Conclusions:**

In this retrospective study, there was no significant difference between the two groups, which may be attributable to the small sample size. A potential advantage of the FQ/ST group might become apparent with a larger cohort. Further research should employ large sample sizes with rigorous adjustment for patient characteristics.

**Disclosures:**

All Authors: No reported disclosures

